# Elevated risk of incarceration among street-involved youth who initiate drug dealing

**DOI:** 10.1186/s12954-016-0120-3

**Published:** 2016-11-22

**Authors:** Carly Hoy, Brittany Barker, Jackie Regan, Huiru Dong, Lindsey Richardson, Thomas Kerr, Kora DeBeck

**Affiliations:** 1 Urban Health Research Initiative, British Columbia Centre for Excellence in HIV/AIDS, St. Paul’s Hospital, 608-1081 Burrard Street, Vancouver, BC V6Z 1Y6 Canada; 2 Interdisciplinary Studies Graduate Program, University of British Columbia, Vancouver, BC Canada; 3 Department of Sociology, University of British Columbia, Vancouver, BC Canada; 4 Division of AIDS, Department of Medicine, University of British Columbia, Vancouver, BC Canada; 5 School of Public Policy, Simon Fraser University, Vancouver, BC Canada

**Keywords:** Drug dealing, Street-involved youth, Incarceration, Employment

## Abstract

**Background:**

Street-involved youth are known to be an economically vulnerable population that commonly resorts to risky activities such as drug dealing to generate income. While incarceration is common among people who use illicit drugs and associated with increased economic vulnerability, interventions among this population remain inadequate. Although previous research has documented the role of incarceration in further entrenching youth in both the criminal justice system and street life, less is known whether recent incarceration predicts initiating drug dealing among vulnerable youth. This study examines the relationship between incarceration and drug dealing initiation among street-involved youth.

**Methods:**

Between September 2005 and November 2014, data were collected through the At-Risk Youth Study, a cohort of street-involved youth who use illicit drugs, in Vancouver, Canada. An extended Cox model with time-dependent variables was used to examine the relationship between recent incarceration and initiation into drug dealing, controlling for relevant confounders.

**Results:**

Among 1172 youth enrolled, only 194 (16.6%) were drug dealing naïve at baseline and completed at least one additional study visit to facilitate the assessment of drug dealing initiation. Among this sample, 56 (29%) subsequently initiated drug dealing. In final multivariable Cox regression analysis, recent incarceration was significantly associated with initiating drug dealing (adjusted hazard ratio = 2.31; 95% confidence interval (CI) 1.21–4.42), after adjusting for potential confounders. Measures of recent incarceration lagged to the prior study follow-up were not found to predict initiation of drug dealing (hazard ratio = 1.50; 95% CI 0.66–3.42).

**Conclusions:**

These findings suggest that among this study sample, incarceration does not appear to significantly propel youth to initiate drug dealing. However, the initiation of drug dealing among youth coincides with an increased risk of incarceration and their consequent vulnerability to the significant harms associated therein. Given that existing services tailored to street-involved youth are inadequate, evidence-based interventions should be invested and scaled up as a public health priority.

## Background

Prior research of street-involved youth indicates that they are a population vulnerable to economic hardship and poor health outcomes [1–5]. This group is highly susceptible to various health-related harms due to problematic substance use, high-risk sexual behaviour, poverty, neglect, and homelessness and housing instability [3, 6, 7]. High-risk behaviours in this population are often compounded by mental health issues, incomplete education, and a lack of marketable job skills, all of which lead to a dependency on the “street economy” [4, 8]. The street economy refers to informal or prohibited income-generating activities that street-involved youth may rely on to meet their basic needs; these activities include but are not limited to panhandling, recycling, acquisitive crime, sex work, and drug dealing [4, 8].

Drug dealing, sex work, “binning” (salvaging recyclable materials), and panhandling are frequently listed as income sources among street-involved youth in urban centres [4, 9, 10]. Drug dealing has been described as the most prevalent prohibited income source among street-involved youth [11], with prior research finding that 58% of this population reported having been involved in the drug trade in the preceding 6 months [12]. Prior research among adult drug-using populations has demonstrated that maintaining personal drug use is the most frequently cited factor underpinning an individual’s initiation into drug dealing [13, 14]. Similar research involving youth who engage in this prohibited income-generating activity suggests that youth are also frequently driven by personal consumption needs [12].

Economic vulnerability is another contributing factor for street-involved youths’ involvement in drug dealing. Previous studies have found that street-involved youth initiate drug dealing for the purpose of fulfilling financial needs that have not been met though legal means [8, 15, 16]. Youth are especially vulnerable to becoming involved in the street economy when their basic survival needs, such as food, clothing, and shelter, are unmet [8]. Further, street-involved youth are frequently unable to rely on the financial support of their families due to estrangement and low socioeconomic status and, as a result, live in situations characterized by extreme income and material insecurity [8, 10]. While a number of health and social services exist with the mandate of addressing some of these vulnerabilities, a recent analysis in our study setting found that more than 60% of street-involved youth reported experiencing difficulty accessing one or more of these services in the last 6 months (e.g. housing, food bank, employment services) [17].

A number of sociodemographic factors exacerbate entrance into street life and associated vulnerabilities. For example, previous research has found that sexual minority youth are overrepresented among street populations, often stemming from stigma and rejection of their sexual orientation at home [8]. Similarly, particularly in Canada, indigenous individuals are disproportionately represented among the homeless and unstably housed [18]. This overrepresentation is present in the criminal justice system as well. A previous study in Vancouver found indigenous street-involved youth were significantly more likely to be incarcerated, despite adjusting for risk factors associated with incarceration, suggesting the possible presence of institutional discrimination within the criminal justice system [19].

As street-involved youth commonly come from backgrounds of trauma and abuse, and incomplete education and may lack strong social ties, these factors exacerbate their precarious economic situations [8, 10]. Previous research has found that vulnerable and street-involved youth with few social ties are more likely to be recruited into the street economy by predatory adults and peers who may pose as mentors or protectors, but in reality are exploiting social and structural vulnerabilities [8]. Lastly, the degree of familiarity and ease by which many street-involved youths can access the street economy makes it an attractive option to meet financial needs [8].

Currently, there are limited efforts aimed at providing economic opportunities for street-involved youth and even less that are sufficiently low barrier to be compatible with their transient lifestyles [1, 20]. A previous study among adult injection drug users in Vancouver found that nearly half of the sample who used and sold drugs would be willing to forgo drug dealing in favour of a low-threshold employment opportunity [1], suggesting that barriers to formal work may be a driving factor. “Low-threshold” work refers to legitimate and low-barrier employment options that may not require adherence to regular schedules, abstinence from drug and alcohol use, and/or accommodate health and social service utilization [1, 11, 21].

Transitions into drug dealing have been found to be associated with other risky behaviours. For example, previous research has found that initiating drug dealing among people who use illicit drugs was associated with dangerous drug use practices, including higher frequency of consistent use, drug “binges”, and the risk of overdose as supply issues become less of a barrier [14, 22]. Both injection and high-intensity drug use have been shown to place individuals at an elevated risk for acquiring infectious diseases such as hepatitis C and human immunodeficiency virus [3, 7, 14]. Furthermore, drug dealing is commonly associated with other risky practices including involvement in organized crime, carrying weapons, violence, and even homicide [9, 23]. These risks have the potential to lead to increased involvement with the criminal justice system and incarceration [23].

The conditions surrounding incarceration have been associated with high rates of depression, psychological disorders, low self-esteem, suicidal ideation, self-harm, and an elevated risk of overdose upon release [24, 25]. Further, evidence suggests that the incarceration period itself has an immensely negative impact on mental health generally and contributes to the onset of mental illness or the worsening of existing pathology [26]. In conjunction with these mental health concerns, transitioning out of prison frequently puts youth at-risk for a variety of economic, social, and physical health-related harms [24, 27–29]. The experience of incarceration has been well documented as a contributing factor in recidivism and further entrenchment of individuals within the criminal justice system [30–33]. However, less is known about whether recent incarceration predicts or is associated with initiating drug dealing among vulnerable youth. Given the well-established harms of incarceration, this longitudinal analysis investigates the relationship between recent incarceration and initiation into drug dealing among street-involved youth in Vancouver, Canada.

## Methods

Data for this study were obtained from the At-Risk Youth Study (ARYS), which is an open prospective cohort of street-involved youth in Vancouver, Canada. To be eligible for the study, participants had to be between the ages of 14–26 years at study enrolment; street-involved, defined as being homeless, unstably housed, or having used a service specific to street-involved youth; to have used an illicit drug other than, or in addition to, marijuana in the past 30 days; and provided written informed consent. This study has been described extensively elsewhere [34]. In brief, at the baseline study visit and biannually thereafter, participants complete an interviewer-administered questionnaire, eliciting a range of information, including sociodemographic information, drug use patterns, sexual and drug-related risk behaviours, engagement with health and social services, and involvement in the criminal justice system. After each visit, participants are remunerated $30 (CAD) for their time. The Providence Health Care/University of British Columbia’s Research Ethics Board has approved the study.

The study period for this analysis extended from September 2005 to November 2014. Data from all participants who reported no history of drug dealing at enrolment and who returned for at least one subsequent study follow-up during the study period to assess for drug dealing initiation were eligible for this analysis. Our primary outcome of interest was initiation into drug dealing, defined as exchanging illicit drugs for monetary reward and including any middle-man activities for monetary reward. The primary explanatory variable of interest was incarceration, defined as responding affirmatively to the question: “Have you been in detention, prison or jail in the last six months?” Participants who reported being incarcerated were then asked to specify the type of facility (e.g. juvenile detention, local jail, provincial, or federal prison) and the number of days incarcerated. As we have done with other outcomes, such as injection initiation and HIV infection [35, 36], the date of when a participant initiated drug dealing was estimated as the midpoint between the first study visit with no reported drug dealing and the follow-up visit where the participant reported engaging in drug dealing. Participants who did not initiate drug dealing throughout the study period were right-censored at the date of their last follow-up visit.

To better understand the potential relationship between drug dealing initiation and incarceration, we adopted a confounding model building approach. Potential confounders were chosen based on a known or hypothesized association with incarceration and drug dealing initiation [12, 14, 37, 38]. These included the following: age (per year older); gender (female vs. male); ethnicity (Caucasian vs. others); homelessness [defined as having no fixed address, sleeping on the street, couch surfing, or staying in a shelter or hostel (yes vs. no)]; any injection and non-injection crystal methamphetamine use (yes vs. no); any crack-cocaine smoking (yes vs. no); any injection and non-injection heroin use (yes vs. no); any injection and non-injection cocaine use (yes vs. no); accessing services [defined as recently accessing any health or social service (e.g. meal programme, supervised injection facility) (yes vs. no)]; being a recent victim of violence [defined as being attacked, assaulted, or suffering violence (yes vs. no)]; ever experiencing physical abuse (yes vs. no); and ever experiencing sexual abuse (yes vs. no). Physical and sexual abuse variables refer to lifetime circumstances. All other drug use and behavioural variables refer to circumstances over the previous 6 months and were treated as time-updated covariates on the basis of semi-annual follow-up data.

To examine the relationship between initiating drug dealing and incarceration, as a first step, we calculated the incidence rate ratio and 95% confidence interval for drug dealing initiation among the sample using a Poisson model. We further examined the baseline characteristics, stratified by reports of initiating drug dealing over study follow-up, and then compared the results using logistic regression. Then, using an extended Cox model with time-dependent variables, we estimated the unadjusted relative hazards and 95% confidence intervals for each explanatory variable that was hypothesized to be associated with drug dealing [39]. The inclusion of time-updated covariates in an extended Cox model negates the requirement of the proportional hazard assumption [39]. To fit our multivariable Cox models, we used a previously described backwards selection process [40, 41], whereby all explanatory variables found to be significantly associated with time to drug dealing initiation in bivariable analyses (*p* value <0.10) were included in the full model. Using a stepwise approach, we subsequently generated a series of reduced models by removing each secondary explanatory variable, one at a time. For each of these models, we assessed the relative change in the coefficient for having been incarcerated in the previous 6 months. The secondary explanatory variable of interest that resulted in the smallest absolute relative change in the coefficient for having been incarcerated was then removed. Secondary variables continued to be removed through this process until the smallest relative change in the coefficient for the effect of incarceration on initiation into drug dealing was observed to exceed 5%. Remaining variables were considered confounders and were included in the final multivariable model. Several authors have previously used this technique successfully [42, 43]. Lastly, to measure if incarceration was a predictor of initiating drug dealing, we lagged accounts of recent incarceration to the previous study visit before participants reported initiating drug dealing. All statistical analyses were performed using SAS software version 9.4 (SAS, Cary, NC, USA). All tests of significance were two-sided.

## Results

Between September 2005 and November 2014, 1175 street-involved youth were recruited into the ARYS cohort. Among 1172 youth enrolled, only 194 (16.6%) were drug dealing naïve at baseline and completed at least one additional study visit to facilitate the assessment of drug dealing initiation. Among this group, during the study period, the average yearly loss to follow-up rate was 2.96%. There were no significant differences with respect to gender (*p* = 0.205), ethnicity (*p* = 0.695), or history of incarceration (*p* = 0.427) between the 194 youth who represented the eligible study sample and the 78 drug dealing naïve youth who were ineligible because they were not enrolled in the cohort long enough to be due for a study follow-up or did not have a follow-up visit at the time this analysis was conducted.

Among the sample of 194 youth included in the study, 91 (47%) were female, 127 (65%) were of Caucasian ethnicity, and the median age was 21 (interquartile range [IQR] 19–23). These 194 youths contributed to 849 observations over the study period. The median number of study visits was 3 (IQR 2–5), the median time between study visits was 5.9 months (IQR 4.9–7.2), and the median follow-up time per participant was 19.7 (IQR 10.8–34.2) months. Over the course of study follow-up, 56 (29%) participants initiated dealing for an incidence density of 13.0 cases per 100 person years (95% confidence interval [CI] 9.9–17.2). At some point during the study period, 55 (28%) youth reported being recently incarcerated. Among those who reported being recently incarcerated, there were 2 (0.2%) reports of being held in a youth detention centre, 49 (5.8%) reports of being held in a local jail, 29 (3.49%) reports of being held in a provincial jail, and 1 (0.1%) report of being held in a federal prison, and the median length of stay was 3 days (IQR 1–18) (it should be noted that youth may have reported multiple incarcerations over the study period and that the percentages reflect the proportion of total observations). Similarly, over the study period, service uptake remained relatively consistent. For example, there were 467 (55.0%) reports of accessing drop-in centres, 246 (29%) reports of accessing an outreach worker, 299 (35.2%) reports of accessing a meal programme, and 111 (13.1%) reports of accessing the supervised injection facility. Table 1 provides baseline characteristics stratified by initiation into drug dealing.

**Table 1:** Baseline sociodemographic characteristics and substance use behaviours associated with initiating drug dealing among street-involved youth in Vancouver, Canada (*n* = 194)

Characteristic	Drug dealing initiation			*p* value
	Yes 56 (29%)	No 138 (71%)	Odds ratio (95% CI)	
Incarceration^a^				
Yes	4 (7)	10 (7)	0.99 (0.30–3.30)	0.985
No	51 (91)	126 (91)		
Age (median, IQR^b^)	21 (19–22)	21 (19–23)	0.94 (0.84–1.05)	0.278
Female gender				
Yes	19 (34)	72 (52)	0.47 (0.25–0.90)	0.022
No	37 (66)	66 (48)		
Caucasian ethnicity				
Yes	36 (64)	91 (66)	0.93 (0.49–1.78)	0.826
No	20 (36)	47 (34)		
Homeless^a^				
Yes	33 (59)	91 (66)	0.76 (0.40–1.45)	0.401
No	22 (39)	46 (33)		
Heroin use^a^				
Yes	13 (23)	45 (33)	0.63 (0.31–1.30)	0.211
No	42 (75)	92 (67)		
Crack-cocaine use^a^				
Yes	24 (43)	66 48)	0.83 (0.44–1.56)	0.569
No	31 (55)	71 (51)		
Crystal methamphetamine used				
Yes	23 (41)	69 (50)	0.71 (0.38–1.33)	0.285
No	32 (57)	68 (49)		
Cocaine use^a^				
Yes	17 (30)	51 (37)	0.76 (0.39–1.49)	0.428
No	38 (68)	87 (63)		
Victim of violence^b^				
Yes	19 (34)	52 (38)	0.90 (0.47–1.75)	0.761
No	34 (61)	84 (61)		
Physical abuse				
Yes	46 (82)	111 (80)	1.19 (0.50–2.86)	0.695
No	8 (14)	23 (17)		
Sexual abuse				
Yes	44 (79)	86 (62)	2.40 (1.11–5.21)	0.026
No	10 (18)	47 (34)		
Accessed services				
Yes	45 (80)	116 (84)	0.78 (0.35–1.73)	0.535
No	11 (20)	22 (16)		

All column percentages may not sum to 100% due to missing data or rounding error

*CI* confidence interval

^a^Denotes activities in the last 6 months

^b^ *IQR* interquartile range

Table 2 shows the unadjusted and adjusted relative hazard of explanatory variables associated with drug dealing initiation. In bivariable Cox regression analysis, incarceration was significantly associated with initiating drug dealing (hazard ratio [HR] = 3.85; 95% CI 2.19–6.76). In multivariable Cox regression analyses, incarceration (adjusted hazard ratio [AHR] = 2.31; 95% CI 1.21–4.42) remained significantly and positively associated with initiating drug dealing after adjusting for the following identified confounders: gender, crystal methamphetamine use, homelessness, and ever experiencing sexual abuse. In order to see if recent incarceration was predictive of drug dealing, we checked for a reverse relationship by lagging incarceration to the previous 6-month study period. However, the bivariable association was not significant (HR = 1.50; 95% CI 0.66–3.42).

**Table 2:** Bivariable and multivariable Cox regression analyses of drug dealing initiation among street-involved youth in Vancouver, Canada (*n* = 194)

Characteristic	Unadjusted hazard ratio		Adjusted hazard ratio	
	HR (95% CI)	*p* value	AOR (95% CI)	*p* value
Incarceration				
Yes vs. no	3.85 (2.19–6.76)	<0.001	2.31 (1.21–4.42)	0.011
Age				
Per year older	0.95 (0.86–1.04)	0.252		
Gender				
Female vs. male	0.53 (0.30–0.93)	0.025	0.54 (0.27–1.08)	0.082
Caucasian ethnicity				
Yes vs. no	0.95 (0.56–1.62)	0.857		
Homeless^a^				
Yes vs. no	2.35 (1.33–4.16)	0.003	1.83 (1.01–3.32)	0.047
Heroin use^a^				
Yes vs. no	2.19 (1.26–3.79)	0.006		
Crack cocaine use^a^				
Yes vs. no	2.87 (1.67–4.93)	<0.001		
Crystal methamphetamine use^a^				
Yes vs. no	2.78 (1.63–4.76)	<0.001	2.60 (1.46–4.62)	0.001
Cocaine use^a^				
Yes vs. no	2.06 (1.18–3.58)	0.011		
Victim of violence^a^				
Yes vs. no	2.00 (1.15–3.47)	0.014		
Physical abuse				
Yes vs. no	1.21 (0.62–2.34)	0.579		
Sexual abuse				
Yes vs. no	2.26 (1.17–4.36)	0.015	2.48 (1.20–5.14)	0.014
Accessed services^a^				
Yes vs. no	1.93 (0.92–4.03)	0.080		

*CI* confidence interval

^a^Refers to activities in the previous 6 months

## Discussion

Among street-involved youth in our sample, drug dealing was highly prevalent at baseline (77%) and subsequent initiation into drug dealing over study follow-up was also high (29%). The overwhelmingly high prevalence of participants with a history of drug dealing at their baseline study visit should be emphasized and underscores the importance of the illicit drug trade to meet this population’s needs. In multivariable analysis with time-updated measures, we found that recent incarceration was positively and significantly associated with initiating drug dealing. While previous research has found similarly significant associations between drug dealing and incarceration [20], our study sought to determine whether dealing predicted incarceration. However, when incarceration was lagged to the study interview prior to the report of initiating drug dealing, the association between incarceration and drug dealing initiation was no longer significant. Therefore, these findings indicate that incarceration does not clearly predict future drug dealing among this sample. Rather, study findings appear to suggest the initiation of drug dealing coincides with an increased risk for incarceration among our study sample.

This interpretation is aligned with previous research indicating that novice, low-level drug dealers are more vulnerable to detection and incarceration [20]. Given the known harms of incarceration, it is evident that further measures should be in established to reduce youth involvement with criminal justice system. Conversely, in 2007, Canada’s federal government implemented the National Anti-Drug Strategy that included Manditory Minimum Sentancing (MMS) legislation [44]. Previous reports have found MMS to be ineffective in reducing population rates of drug dealing, as it targets low-level, street drug dealers who are primarily involved in dealing to support personal drug use [45]. As many youths are early in their drug dealing careers and dealing to mitigate their economic insecurity, an unintended consequence of MMS is that youth may be more susceptible to being arrested and incarcerated.

Our finding of a significant association between recent drug dealing initiation and incarceration suggests a need for evidence-based interventions to improve the economic security of youth and prevent them from resorting to drug dealing. A study based in Los Angeles found that low-threshold social enterprise employment interventions were effective in utilizing street-involved youths’ existing entrepreneurial skills that may have been developed through their experience with the street-based economy, such as management and budgeting [46]. The programme taught and strengthened complementary skills necessary to obtain legitimate means of employment as an alternative to the street-based economy, and many of the youth involved in the programme have transitioned into long-term employment in a variety of industries [46]. Low-threshold employment programmes aimed at addressing underlying barriers to employment such as low self-esteem, incomplete education, substance use, and homelessness have also been shown to be effective among street-involved youth [47]. For example, a youth employment programme in northern British Columbia offered part-time employment opportunities to vulnerable youth with the intention of moving youth into full-time employment if they were able to work with staff to address their individual barriers to employment [47]. Eighty-eight percent of youth who completed the programme were able to find either formal employment or enrol in school at follow-up a year later [47]. Another example, located in the present study’s setting, offers a low-threshold employment programme, where street-involved youth can work casual shifts to assist with “street beautification” and are compensated with a financial honorarium on the same day [48]. The extent to which low-threshold interventions of this nature successfully address the economic vulnerabilities experienced by street-involved youth and may subsequently reduce engagement in drug dealing and risk of incarceration warrants rigorous evaluation as the potential for public health benefits are significant.

For youth who do become involved with the criminal justice system, ensuring there are viable economic opportunities post-release has the potential to address vulnerabilities previously mentioned. For example, a meta-analysis conducted in 2009 found that engagement with counselling, multiple coordinated services (e.g. case management, residential services), skill building, and restorative interventions (e.g. restitution and mediation between offenders and victims) had a moderate impact on reducing youth recidivism (between 10 and 13%) [49]. Additionally, a study conducted in 2004 among adult offenders with a history of drug use found that post-release employment programmes had a high programme completion rate (78%), and more than half secured competitive employment, often including benefits [50].

This study has limitations. First, as with all community-recruited research cohorts, the ARYS cohort is not a random sample and therefore may not be generalizable to other populations of street-involved youth. Second, data collected was based on self-report and thus could be subject to response bias, including socially desirable responding, which may have resulted in the under-reporting of illicit substance use, engagement in drug dealing, and other stigmatized activities. However, self-reported risk behaviour has been shown to be largely accurate among adult substance-using populations [51], as well as among various youth populations [52]. Furthermore, the non-randomized nature of this study results in the conclusions being potentially influenced by confounders that are not accounted for. Given the extremely high prevalence of drug dealing at baseline, future research should investigate the relationship between incarceration and initiating drug dealing using larger samples. Further, our broad definition of incarceration in the current study (e.g. detained, charged, prosecuted) may result in the estimates being under- or overestimated. Future studies that differentiate between distinct types of criminal convictions and lengths of incarceration events would be beneficial. Additionally, our study instrument did not measure the reason youth were incarcerated. As a result, we are unable to assess if incarceration events were related to drug dealing vs. violent crime, acquisitive crime, or other types of offences. Therefore, it should be noted that income-generating crimes may not be the only activity related to incarceration in this population. Future research in this area would be helpful to better inform the relationship between the initiation of drug dealing and incarceration.

## Conclusions

In summary, among our study sample of street-involved youth, we found that the experience of recent incarceration does not appear to significantly drive youth to initiate drug dealing. However, the initiation of drug dealing was found to coincide with an elevated risk for incarceration. This finding suggests that efforts to improve the economic security of street-involved youth may have the potential to reduce engagement in drug dealing and subsequent harms including incarceration. Given the inadequacy of existing services among this population, governments should invest and scale up evidence-based interventions to help reduce the economic vulnerabilities experienced by street-involved youth.

## Abbreviations

ARYS: At-Risk Youth Study
MMS: Mandatory Minimum Sentencing
